# Development and psychometric properties of the conscience-based nursing care scale

**DOI:** 10.3389/fpsyg.2025.1507647

**Published:** 2025-03-11

**Authors:** Homeira Khoddam, Abbas Ebadi, Mahnaz Modanloo, Soheyla Kalantari

**Affiliations:** ^1^Nursing Research Center, Golestan University of Medical Sciences, Gorgan, Iran; ^2^Nursing Care Research Center, Clinical Sciences Institute, Baqiyatallah University of Medical Sciences, Tehran, Iran; ^3^Surgical Technology Department, Laboratory Science Research Center, Golestan University of Medical Sciences, Gorgan, Iran

**Keywords:** laboratory science research center, surgical technology department, Golestan conscience, nursing care, scale, mixed method study

## Abstract

**Background:**

It is clear that nurses’ conscience plays an important role in guiding professional decision-making and ensuring quality patient care. Additionally, it positively impacts nursing performance and promotes ethical care in clinical settings.

**Aims:**

This study aimed to develop and validate the conscience-based nursing care scale (CNS) based on the clinical nurse care setting in Iran.

**Methods:**

This study is a sequential exploratory mixed-methods study. The concept of “conscience-based care” was clarified using Schwartz-Barcott and Kim’s hybrid concept analysis method, which included a comprehensive literature review and qualitative fieldwork involving in-depth interviews with five nurses. Then, the psychometric properties of a newly developed scale, based on the themes identified in the first phase, were evaluated. This included item generation, face and content validity assessments, a pilot study, and both exploratory and confirmatory factor analyses.

**Results:**

The developed scale has 24 items spread across five factors, namely accountability in conscientious care, responsibilities in care, attention to the quality of care and teamwork, importance to the reputation and dignity of the profession, and ethics in care. For each item, five options (always, most of the time, sometimes, rarely, and never) were considered, with the numerical value of each being always = 5, most of the time = 4, sometimes = 3, rarely = 2, and never = 1 determined based on the meaning of the item. Exploratory and confirmatory factor analyses confirmed its structure, explaining 43.79% of the variance in conscience-based care. The scale demonstrated high reliability (ICC = 0.959) and responsiveness to change, with minimal ceiling and floor effects.

**Conclusion:**

This study validated the CNS in an Iranian clinical setting, demonstrating its reliability and validity. Nursing managers and policymakers can confidently use this scale to assess the quality of nursing care and encourage nurses to provide conscience-based care.

## 1 Introduction

The concept of care has been defined as a goal, mission, essence, and ethical ideal for guiding the nursing practice (Meleis, 2011). It should be noted that only the knowledge and clinical performance of a nurse in care are not enough, but the patient needs various levels of ethical and psychological care for complete improvement (Bayattork et al., 2019; Meleis, 2011). Nurses analyze care issues in clinical situations from an ethical perspective and use their reasoning and decision-making abilities to provide appropriate ethical care (Schuklenk, 2019).

Conscience is the foundation and cornerstone of ethical care in nursing practice (Cleary and Lees, 2019; Mohamadi et al., 2019) and defines nurses’ personal integrity, beliefs, and values (Kukla, 2002; Lewis-Newby et al., 2015). It is clear that the nurses’ conscience plays an important role in guiding professional decision-making and quality of patient care, has a positive impact on nursing performance, and promotes ethical care in clinical settings (Gulec and Aslan, 2024).

Jensen and Lidell (2009) describe conscience as a motivating factor for nurses to perform courageous care actions. Backed by conscience-based courage in clinical performance, nurses carry out actions based on their own experience and knowledge in a professional manner. In a clinical setting, conscience can serve as an alert system indicating nurses’ personal and professional values, ethical beliefs, and standards that are threatened by dilemmas and challenges in various situations.

When nurses are asked to recount ethical problems while caring for patients, they often rely on their conscience to stop them from performing some actions and motivate them to perform other actions (Jasemi et al., 2019a). In some cases, nurses use conscientious objection when faced with challenging ethical and moral situations. Situations such as different approaches of colleagues and organizations regarding caring for dying patients, refusal of treatment, refusal of blood transfusion, or voluntary termination of a fetus by parents can lead to nurses’ conscientious objection, causing internal conflict as they navigate their own conscience in the workplace (Toro-Flores et al., 2019).

In a study by Lamb and Pesut (2021), nurses acknowledged that they had moral and conscientious objections to addressing ethical and moral issues present in clinical practice. The results of another study showed that 57% of nurses with conscientious objections were forced to leave their profession due to insufficient staffing, failure to uphold patient rights, and being required to care for patients contrary to their beliefs (Zampas and Andión-Ibanez, 2012).

The concept of conscience is associated with culture and society (Jasemi et al., 2019b). The definition of professional conscience in one cultural context will be different from another culture and society (Toro-Flores et al., 2019). Therefore, it is necessary to first provide an accurate measurement of conscience-based care, based on which the conditions and situations in providing conscience-based care can be determined. Undoubtedly, this measurement scale should be designed based on the cultural context of the society in question to provide an appropriate reflection of the current situation. In this study, an effort has been made to first conceptualize the structure of conscience-based care and design a scale for measuring this concept in the context of Iranian clinical setting and then to conduct its validation process in a methodological manner.

## 2 Method

This study adopts a sequential exploratory mixed-methods approach. Conscience-based care was conceptualized using a hybrid concept analysis method. Following the qualitative phase and theme extraction, the psychometric properties of the scale were evaluated.

### 2.1 Conceptualization

The study aimed to clarify the concept of “conscience-based care” in nursing using Schwartz-Barcott and Kim (2000) hybrid model, which includes theoretical, fieldwork, and final analysis stages. Initially, a comprehensive literature review was conducted to develop an operational definition, resulting in the selection of 45 relevant articles. In the fieldwork stage, in-depth interviews with five nurses from various clinical departments in northern Iran were conducted to refine and culturally contextualize the concept. For performing content analysis, the participation of at least five participants is adequate (Kalantari et al., 2024). Data from these interviews were analyzed using qualitative content analysis. Finally, insights from both the literature review and fieldwork were integrated to solidify the concept, laying the groundwork for designing a measurement scale (Kalantari et al., 2024).

### 2.2 Scale design and psychometric evaluation

#### 2.2.1 Item generation

Based on the concept mapping phase of the analytical concept, which was based on the concept features in the theoretical and fieldwork phases, a pool of 105 items was formed. After reevaluation, items that were redundant and similar were removed, and ultimately 87 items were entered into the psychometric evaluation phase.

#### 2.2.2 Face validity

To evaluate face validity, both quantitative and qualitative methods were used. Initially, a quantitative content validity method was applied using the item impact approach. To ensure a proper understanding of the items, 10 nurses were selected. In this stage, maximum diversity in terms of individual and professional characteristics was considered for selecting participants. Participants were asked to rate each item on a fully understandable Likert scale ranging from 5 (completely understandable) to 1 (completely incomprehensible).

The researcher calculated the impact score of each item separately using the following formula: Frequency (%) × Importance. Frequency represents the number of individuals who assigned a score of 4 and 5 to each item, while comprehension represents the ability to understand the item based on the Likert scale (Behboodi Moghadam et al., 2024).

Items that scored less than 1.5 entered the stage of qualitative validity. In this stage, the relevant items were examined through face-to-face interviews in terms of ambiguity and clarity. Finally, items that needed correction were modified to increase the respondents’ perception.

### 2.3 Content validity

The content validity of CNS was evaluated both qualitatively and quantitatively. The qualitative content validity of CNS was determined by asking 18 experts with clinical and scientific expertise in nursing ethics and scale development to evaluate the items’ words, grammar, item allocation, scoring, and scaling. To assess the validity of the content quantitatively, the scale was given to 18 experts and the indicators of the content validity ratio (CVR) in terms of essentiality, the content validity index (CVI), and modified kappa index (K*) was measured in terms of relevance (Polit et al., 2007).

For evaluating CVR, each item was examined based on a three-part Likert scale: (1 = not essential, 2 = useful but not essential, 3 = essential). According to the Lawshe formula with the number of 18 experts, the minimum acceptable amount of CVR is 0.45. If the resulting score is greater than 0.45, the validity of the content of that item is confirmed; otherwise, it will be removed (Mokhtarinia et al., 2021).

Following this, modified Kappa (K*), as recommended by Polit et al. (2007), was calculated by asking 18 experts to rate each item. To calculate K*, the probability of chance agreement was first calculated using the following formula: PC = [*N*!/(*A*! (*N* – *A*)!)] × 0.5 *N*, where *N* is the total number of raters and *A* is the number of agreements regarding the item relevance. Eighteen experts evaluated the relevancy of each item of the CNS using a 4-point Likert scale (1 = not relevant, 2 = somewhat relevant, 3 = relevant, 4 = completely relevant). The item-CVI score (I-CVI) was calculated by dividing the number of experts who gave that item a score of 3 or 4 by the total number of experts. K* was then calculated using the following formula: K* = ((*I* – CVI) – PC)/(1 – PC). A kappa value greater than 0.74 was considered good and acceptable. For scale-CVI (average I-CVIs for the entire scale), a score of 0.80 or higher was favorable (Polit et al., 2007).

#### 2.3.1 Pilot study (item analysis)

In a pilot study with a sample of 50 nurses, internal consistency was assessed using Cronbach’s alpha coefficient and inter-item correlation for decision-making about items before factor analysis. The participants had a mean age of 48.31 years with a standard deviation of 5.91. Thitry seven nurses were women (74%) and 13 were men (26%). Item analysis was conducted to identify potential problems with individual items or the scale as a whole. Items with correlation coefficients less than 0.32 or greater than 0.9 were removed (Delshad et al., 2024). This sample (50 nurses) was not selected for the next phases of the study.

#### 2.3.2 Construct and structural validity

##### 2.3.2.1 Participants

Iranian nurses were selected as participants using the convenience method. The study focused on clinical nurses from the Golestan University of Medical Sciences. According to the rule of thumb, 300 and 250 participants are considered sufficient for exploratory factor analysis (EFA) and confirmatory factor analysis (CFA), respectively (Widaman and Helm, 2023). In order to perform EFA and CFA, a total of 600 nurses were recruited.

##### 2.3.2.2 Measurement scale

**Figure 1:**
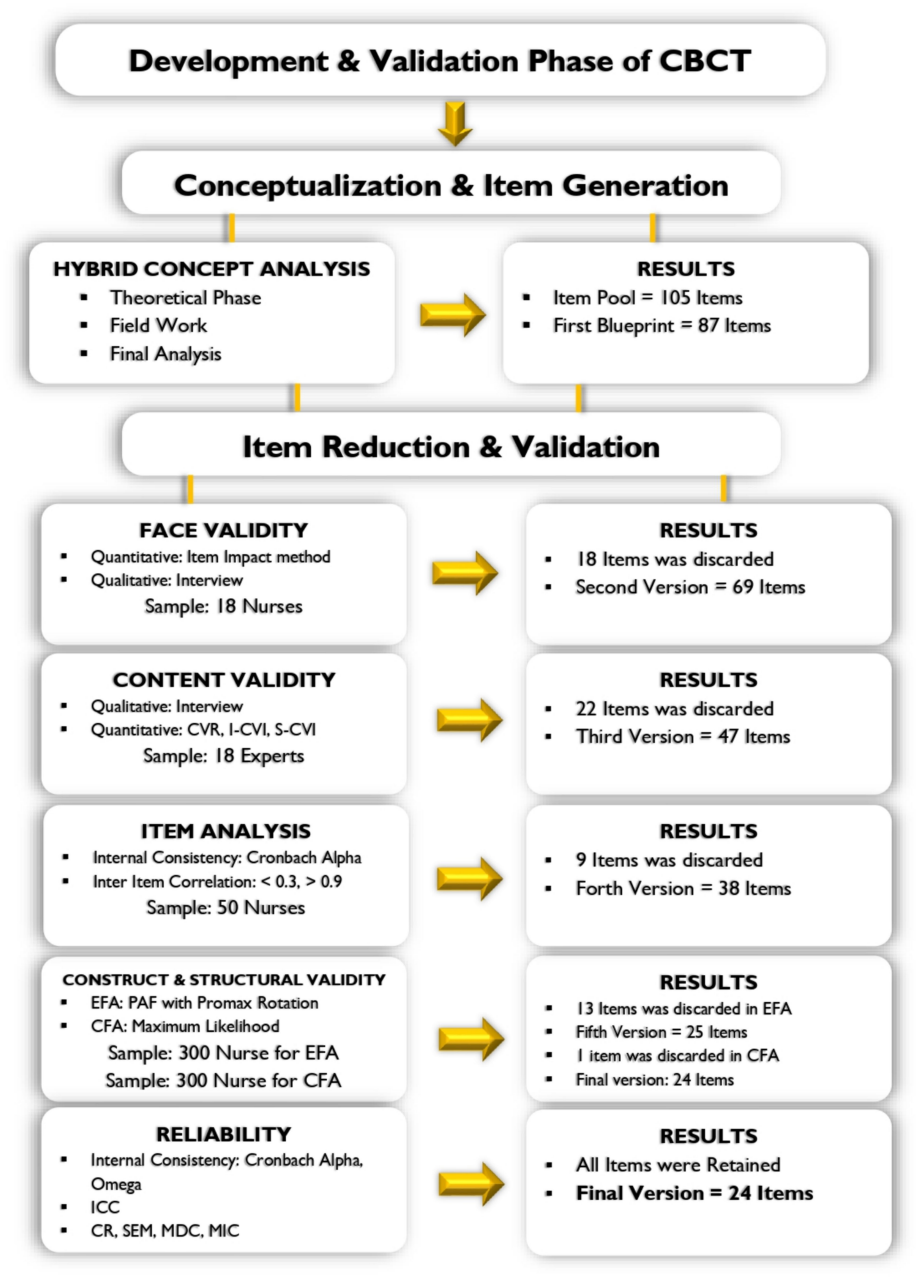
Development and validation phase of CNS.

After refining the scale in the previous stages, a 38-item scale entered the structural validity stage, and an exploratory factor analysis was performed. The item reduction stages are significant as shown in Figure 1.

#### 2.3.3 Exploratory factor analysis

The construct validity of CNS was evaluated by EFA and CFA. The EFA was evaluated through the principal axis factoring (PAF). According to the correlation results of more than 0.3 between factors, Promax rotation, which is the most common rotation used in humanities studies, was used (Teymoori et al., 2024). Kaiser–Meyer–Olkin (KMO) levels above 0.7 were considered to be acceptable. KMO is used in factor analysis to determine if the data is suitable for factor analysis by measuring sampling adequacy (Roshan et al., 2023; Sharif-Nia et al., 2024).

To determine that, the measurement scale under study (or more precisely, the set of items) is saturated by several significant factors. Three major indices were considered: (a) eigenvalue, (b) proportion of variance explained by each factor, and (c) scree plot. In conducting PAF analysis for the 38-item scale, factor loadings above 0.32 were considered important and significant in defining the factors (Widaman and Helm, 2023).

#### 2.3.4 Confirmatory factor analysis

At this stage, the structure of the construct obtained through EFA was examined by CFA. Based on the rule of thumb proposed by Hair et al. (2020), a total of 300 nurses were included in the study using a convenience sampling method. The maximum-likelihood approach was used for the CFA. The model fit with data was evaluated with the help of standard model fit indices. Among the goodness-of-fit indices, the most commonly used were evaluated for the presented model. From the absolute fit indices, the chi-square (CMIN) and root mean square error of approximation (RMSEA < 0.08) were assessed. In the comparative fit indices, the Tucker–Lewis Index (TLI > 0.9), Incremental Fit Index (IFI > 0.9), and Comparative Fit Index (CFI > 0.9) were calculated. For parsimonious fit indices, the Parsimonious Normed Fit Index (PNFI > 0.5) and Parsimonious Comparative Fit Index (PCFI > 0.5) were calculated, along with the ratio of chi-square to degrees of freedom (CMIN/DF < 5) was also evaluated (Bigdeli Shamloo et al., 2023; Sharif-Nia et al., 2023).

#### 2.3.5 Convergent and discriminant validity

The convergent and discriminant validity were assessed using the average variance extracted (AVE), maximum shared squared variance (MSV), and composite reliability (CR). As a result, the criteria for the existence of the convergent validity were AVE > 0.5, CR more than AVE, and MSV less than AVE, while the criteria for the existence of the discriminant validity were MSV less than AVE (Fornell and Larcker, 1981). Additionally, an innovative approach of Henseler’s heterotrait-monotrait ratio (HTMT) criteria was used to assess the discriminant validity. HTMT ratio < 0.85 was considered as the existence of discriminant validity (Henseler et al., 2015).

#### 2.3.6 Reliability

In this study, in order to determine the internal consistency of the structure, CNS was completed using Cronbach’s alpha coefficient, McDonald’s omega coefficient (*Ω*), and average inter-item correlation (AIC). Alpha and Omega coefficients >0.7 and AIC 0.2–0.4 were considered appropriate. Furthermore, the composite reliability (CR) of the final structure, which is the strongest type of reliability evaluation, and the maximum reliability value (MaxR) > 0.7 was considered acceptable (Moreno Jiménez et al., 2014).

In this study, the test–retest method was used to check the relative stability. In this way, 25 nurses were asked to complete the scale and it was repeated 2 weeks later under the same conditions. Then, the agreement between the scores obtained from the two tests was calculated using the intraclass correlation coefficient (ICC) and the two-way random-effects model. The value of this index is higher than 0.8, indicating that reliability is acceptable (Hosseini et al., 2023).

Absolute reliability was evaluated based on standard error of measurement (SEM) by the following formula: SEM = SD Pooled × √(1 − ICC) [30]. The responsiveness was evaluated by calculating the minimal detectable change (MDC) via the formula: MDC95 = SEM × √2 × 1.96 and minimal important change (MIC) via the formula: MIC = 0.5 × SD of the *Δ* score, respectively (Yuan and Kelly, 2019).

The minimum detectable change percentage (MDC%) is calculated as follows to determine the actual relative changes after intervention or repeated measurements over time, as well as to demonstrate the relative amount of random measurement error:


$$MDC\%=\left(MDC÷\mathrm{Mean}\right)\times 100$$

The acceptable minimum detectable change percentage is less than 30%, and a minimum detectable change percentage below 10% is considered excellent. Finally, the ceiling and floor effect as well as MDC were evaluated to determine interpretability (Terwee et al., 2021).

#### 2.3.7 Multivariate normality and outliers

Multivariate outliers were analyzed using Mahalanobis distance (*p* < 0.001), while univariate outliers were evaluated using distribution charts. Additionally, skewness (±3) and kurtosis (±7) were used to evaluate the normality of the univariate distribution, and Mardia’s coefficient < 8 was used to check the normality of the multivariate distribution (Jazi et al., 2020).

#### 2.3.8 Data analysis

The data were analyzed using IBM SPSS Statistics ver.24 /AMOS 24.

#### 2.3.9 Ethical considerations

The study was conducted as part of a PhD dissertation on nursing and was approved by the Ethics Committee of Golestan University of Medical Sciences (code of ethics: REC.GUMS.IR1399.245). The research was conducted in accordance with the principles of the Declaration of Helsinki. Participants were informed of the study’s objectives and they were further assured that participating in the study was voluntary. Participants received assurances of the confidentiality of their data.

## 3 Results

### 3.1 Conceptualization and item generation

#### 3.1.1 Conceptual clarification

In this stage, conceptual clarification was done using a hybrid concept analysis method. The final analysis compared the data from the work stage with the findings from the theoretical stage, leading to the classification in Table 1. Based on the findings and subcategories, as well as main categories, propositions were generated in a way that provides a comprehensive coverage of the clarified concept.

**Table 1:** Explanation of the extracted concept from the analysis of the hybrid concept.

Concept	Main layers	Sublayers
Conscience-based care	Ethics in the heart of conscience-centered care	Moral sensitivity
		Moral courage
	Professional care as conscientious care	Ethical care
		Client-centered
		Humanistic care
	Care with a focus on professional commitment	Professional identity
		Service motivation

#### 3.1.2 Item generation

To generate unique items, 105 items were created in the form of an item pool. These items were discussed and reviewed in multiple research team sessions. For better coverage of each area related to conscience-based care, the items were reviewed several times and after adding, integrating, or changing some items, a total of 87 items were formulated as the initial version. For each item, five options were considered, with the numerical value of each being always = 5, most of the time = 4, sometimes = 3, rarely = 2, and never = 1 determined based on the meaning of the item. None of the items had reverse scoring.

### 3.2 Item reduction

#### 3.2.1 Face, content validity, and item analysis

The results of the quantitative face validity of the scale led to the elimination of 18 items, reducing the total number of items from 87 to 69. In the qualitative content validity phase, 14 items were reviewed. No items were eliminated in this phase but only revised. Based on the results obtained from CVR, 22 items were removed, and the total number of CNS was reduced from 69 to 47 items. Based on the results obtained of Modified Kappa (K*), none of the items were removed. In item analysis, nine items were removed due to not meeting the desired criteria, resulting in the instrument entering the construct validity stage with 38 items (Table 2).

**Table 2:** The result of EFA on CNS (*N* = 300).

Factors	Number: Items	Factor loadings	*h*^2^	λ	% variance
Accountability in conscientious care	Q_31_: In case of crises and natural disasters such as wars, earthquakes, floods, disease epidemics, etc., I volunteer to provide relief	0.767	0.520	5.575	20.165
	Q_28_: I will do my best to take care in such a way that the least harm will not come to others	0.688	0.481		
	Q_30_: Nurses’ salaries and benefits are important to me, but they do not affect the quality of care for my patients	0.669	0.558		
	Q_27_: I am responsible for my professional performance	0.668	0.510		
	Q_33_: I try to take care beyond what is my professional duty	0.556	0.470		
	Q_25_: The patient’s ungratefulness does not affect the provision of correct and accurate care	0.350	0.205		
Responsibilities in care	Q_13_: I keep the secrets of applicants confidentially in special cases	0.882	0.703	3.149	10.594
	Q_10_: By identifying and reporting my professional errors, I prevent possible harm to the patient	0.787	0.626		
	Q_11_: If requested, I will attend the patient’s bedside immediately	0.615	0.509		
	Q_12_: While providing care, I try to make the patient suffer less	0.609	0.369		
	Q_38_: I avoid imposing the pressure of my professional duties on colleagues	0.404	0.286		
Attention to the quality of care and teamwork	Q_22_: Despite the lack of facilities and lack of quality equipment, I try to provide effective and standard care to patients	0.761	0.515	1.763	4.801
	Q_20_: If needed, I get help from my colleagues to fully care for the patient	0.659	0.557		
	Q_23_: I give hope and peace to patients	0.643	0.422		
	Q_21_: My previous experiences help me to make the right decision in different situations	0.532	0.479		
	Q_19_: I help the care team if the workload increases with little time	0.469	0.419		
	Q_18_: I keep my knowledge, attitude and professional performance up-to-date with continuous self-education	0.392	0.238		
	Q_16_: At the time of discharge, I provide complete information verbally	0.372	0.351		
Importance to the reputation and dignity of the profession	Q_35_: I act in such a way that the credibility and dignity of the nursing profession is not questioned	0.714	0.469	1.643	4.274
	Q_34_: I try to manage the situation such as anger and misbehavior of the patient and companions with a favorable and professional approach	0.711	0.526		
	Q_37_: I am active in teaching and guiding novice and less experienced nurses	0.613	0.457		
Ethics in care	Q_5_: When making decisions, I prioritize the values of professional ethics over organizational and personal interests	0.694	0.453	1.518	3.951
	Q_4_: I follow the principles of professional ethics in clinical situations	0.544	0.267		
	Q_3_: In cases where the benefit of the patient is involved, I tolerate any threat from the organization and managers	0.496	0.345		
	Q_8_: In performing clinical care, I consider the respect and dignity of the patient	0.396	0.212		

#### 3.2.2 Construct and structural validity

**Figure 2:**
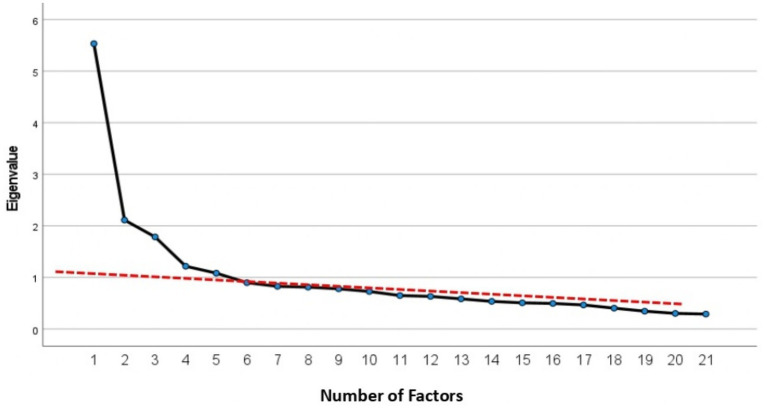
Scree plot and factor number.

In EFA with the principal axis factoring method in 300 samples, KMO (0.806) and Bartlett’s value 2819.77 (*p* < 0.001) showed the sampling adequacy and the absence of an identity matrix. After performing PAF with Promax rotation, five factors were extracted based on the Kaiser criterion and special value. The scree plot also indicated the correctness of the extracted factors (Figure 2). After factor analysis, 13 items were removed due to the lack of significant factor loading on any of the factors.

In the EFA, after applying Promax rotation, five factors with a total number of 25 items were extracted from 38 items. As a result, CNS with 25 items were classified into five factors, namely, “Accountability in conscientious care” with six items, “Responsibilities in care” with five items, “Attention to the quality of care and teamwork” with seven items, “Importance to the reputation and dignity of the profession” with three items, and “Ethics in care” with four items. These five factors explained 43.79% of the total variance of Conscience-Based Care in nurses.

**Figure 3:**
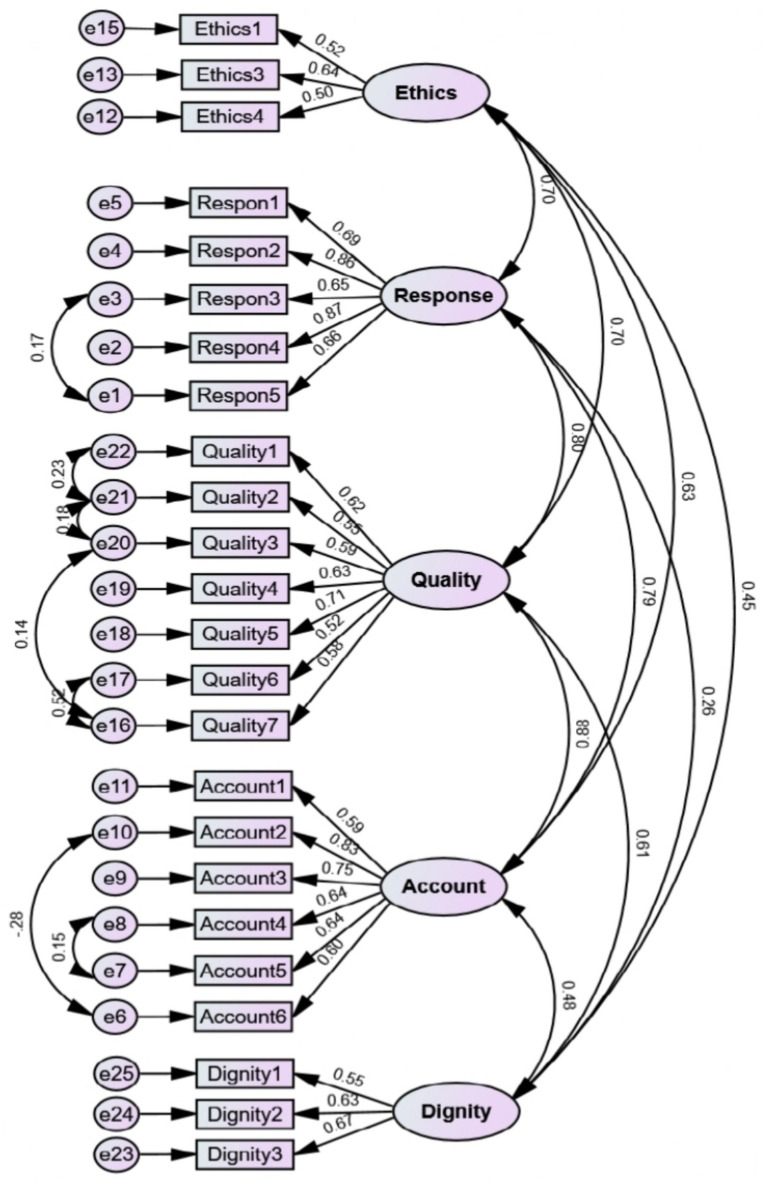
First-order CFA of CNS (*n* = 330).

The structural validity of the model was evaluated through CFA, and the fit indices of the model were found to be within the acceptable range, confirming the model (Figure 3; Table 3). One item in ethics care due to low factor loading was discarded. Therefore, the final scale has 24 items. The scale factors were found to be convergent, and the results of AVE, MaxR(h), and CR analyses were used for convergent validity. All items had discriminant validity, and the results of HTMT showed no warnings for discriminant validity (Tables 4, 5).

**Table 3:** Fit indices of CFA model.

Indices	*Χ*^2^	Df	*p-*_value_	CMIN/DF	RMSEA	PNFI	PCFI	TLI	IFI	CFI
CFI	474.54	235	<0.001	2.03	0.059	0.726	0.782	0.904	0.919	0.918
Acceptance range	–	–	≥0.05	≤3	≤0.08	≥0.5	≥0.5	≥0.9	≥0.9	≥0.9

**Table 4:** Convergent validity of CFA constructs.

Constructs	α	CR	MaxR (H)	AVE
Accountability in conscientious care (account)	0.836	0.828	0.894	0.462
Importance to the reputation and dignity of the profession (dignity)	0.649	0.648	0.858	0.381
Ethics in care (ethics)	0.570	0.576	0.806	0.312
Attention to the quality of care and teamwork (quality)	0.820	0.749	0.654	0.364
Responsibilities in care (response)	0.864	0.858	0.585	0.566

**Table 5:** Discriminative validity of CFA constructs (HTMT criteria).

Constructs	Account	Dignity	Ethics	Quality
Account				
Dignity	0.501			
Ethics	0.639	0.471		
Quality	0.829	0.625	0.684	
Response	0.789	0.294	0.687	0.786

#### 3.2.3 Reliability assessment

In this study, the intraclass correlation coefficient (ICC) was used to determine stability (repeatability) and external homogeneity. The results of the study regarding the ICC showed that the ICC value for the entire scale was 0.959 with a 95% confidence interval of 0.682–0.984, indicating the scale’s stability over time. Details are shown in Table 6.

**Table 6:** Relative stability index (stability) of conscience-based care dimensions.

Factor	α	Ω	ICC	CI (95%)	*p* value
Responsibility in care	0.896	0.897	0.947	0.863, 0.977	<0.001
Conscience-centered care accountability	0.846	0.847	0.942	0.695, 0.980	<0.001
Attention to care quality and teamwork	0.939	0.945	0.975	0.930, 0.982	<0.001
Importance of professional reputation and dignity	0.891	0.897	0.867	0.678, 0.941	<0.001
Ethical care	0.614	0.674	0.816	0.659, 0.928	<0.001
Overall	0.920	0.918	0.959	0.682, 0.984	<0.001

In order to achieve the goal, the reliability and interpretability of the Conscience-Based Care scale were determined in this study. Absolute reliability was examined based on SEM calculation, which had a total value of 3.037. This value indicates that the scale score in an individual item varies by ±3.037 in repeated testing. Since the MIC is smaller than the MDC, it can be concluded that the scale is responsive to changes. As the percentage of detectable changes is less than 30%, the changes between the two test scores are real and the designed scale is capable of detecting minimal changes. The details are shown in Table 7. The ceiling and floor effect in this study was equivalent to 3.47%, which is considered desirable.

**Table 7:** Measurement error indices, minimum detectable change, and minimum clinically important difference in conscience-based care instruments.

Factor	SD_Pooled_	Mean difference	SD difference	SEM^*^	MDC^**^ (%95)	MIC^***^	MDC (%)
Responsibility in care	3.655	0.70	1.35	0.841	2.324	0.675	10
Conscience-centered care accountability	3.284	1.167	1.34	0.790	2.183	0.670	9
Attention to care quality and teamwork	5.222	0.80	1.47	0.130	0.359	0.735	2
Importance of professional reputation and dignity	2.456	0.80	1.56	0.895	2.472	0.780	19
Ethical care	1.729	0.90	1.15	0.741	2.047	0.575	18
Overall	15	4.367	4.32	3.037	8.382	2.16	8

*Standard error of measurement, **Minimal detectable change, ***Minimal important change.

## 4 Discussion

This study aimed to develop and validate the CNS to assess nursing care practices using conscience as the basis within the Iranian clinical context. The final 24-item scale, structured across five factors—accountability in conscientious care, responsibilities in care, attention to the quality of care and teamwork, importance to the reputation and dignity of the profession, and ethics in care—explained 43.79% of the variance in conscience-based care. In the following discussion, these findings are integrated with the existing literature, highlighting their implications, limitations, and areas for future research.

The CNS was validated as a reliable and responsive tool for measuring conscience-based care. The key dimensions identified include accountability in care, which underscores nurses’ commitment to outcomes and responsibility for their actions, and responsibility in care, which emphasizes the avoidance of negligence and the promotion of patient safety. The attention to quality and teamwork dimension highlights the collaborative and patient-centered nature of conscientious nursing care, while the importance to the profession’s reputation underscores the ethical commitment to uphold nursing standards. Finally, ethics in care reflects adherence to moral principles, professional integrity, and respect for patient dignity.

These dimensions collectively capture the essence of conscience-driven nursing care, offering a comprehensive framework to understand how ethical principles are integrated into clinical practice. The tool’s structure facilitates targeted interventions to enhance specific aspects of nursing care, making it a valuable resource for both practice and research.

These findings are consistent with prior research on the role of conscience in nursing practice. The emphasis on accountability aligns with Glasberg et al. (2006), who noted that conscience acts as a regulatory mechanism influencing nurses’ decisions and performance. Similarly, the importance of responsibility in care reflects insights from Yazdanian et al. (2016), which highlight the critical role of duty-oriented behavior in ensuring patient safety. The attention to teamwork and quality underscores the necessity of interdisciplinary collaboration, as emphasized by Jasemi et al. (2019b), who identified teamwork as a cornerstone of ethical nursing care.

The focus on maintaining the profession’s reputation mirrors the findings of Johnson and Bowman (1997), who highlighted the importance of ethical behavior in upholding nursing’s credibility. The ethics in care dimension reflects principles outlined by Lamb and Pesut (2021), which prioritize patient autonomy, confidentiality, and adherence to moral decision-making frameworks. Together, these comparisons reinforce the validity of the CNS dimensions and highlight their alignment with broader nursing ethics literature.

## Limitations

One limitation of this study is the cultural specificity of the CNS. While the scale was rigorously developed and validated within an Iranian context, its applicability in other cultural settings remains to be tested. Ethical care practices are influenced by cultural norms and values, and future research should explore the cross-cultural validity and reliability of the CNS. Comparative studies across different healthcare systems can provide valuable insights into the universal and context-specific aspects of conscience-based care.

Another limitation is the relatively narrow focus on clinical nurses. Expanding the study to include other healthcare professionals, such as midwives and allied health workers, could enhance the scale’s applicability and provide a more comprehensive understanding of conscience-based care in diverse healthcare settings. Additionally, longitudinal studies are needed to assess the scale’s sensitivity to changes in ethical practices over time.

Future research should focus on adapting the CNS for use in other cultural and professional contexts. Studies could explore its application in diverse healthcare settings, including high-pressure environments like intensive care units and emergency departments. Additionally, research could examine the relationship between conscience-based care and patient outcomes, such as satisfaction, safety, and quality of life. Investigating the impact of interventions designed to enhance conscience-based care, such as ethics training and interdisciplinary collaboration programs, would also be valuable.

### 4.1 Implications

The CNS provides a culturally adapted and psychometrically robust tool for assessing conscience-based nursing care. It offers nursing managers and policymakers an evidence-based framework to evaluate and improve the quality of ethical nursing practices. By focusing on key dimensions such as accountability, teamwork, and ethics, healthcare institutions can prioritize training and interventions that foster a culture of conscientious care.

The educational implications of this study are equally significant. Integrating the CNS dimensions into nursing curricula can strengthen students’ understanding of ethical principles and prepare them for the complex moral dilemmas they may encounter in clinical practice. The scale’s emphasis on professional reputation highlights the need to cultivate a sense of pride and responsibility among nurses, encouraging them to act as advocates for their patients and their profession.

## 5 Conclusion

This study confirmed acceptable psychometric properties and the factor structure of the CNS in an Iranian sample. Given these findings, the scale can be used as a valid and reliable scale for the assessment of conscience-based nursing care by Iranian nurses.

## Data Availability

The raw data supporting the conclusions of this article will be made available by the authors, without undue reservation.
